# Performance optimization of a microwave-coupled plasma-based ultralow-energy ECR ion source for silicon nanostructuring

**DOI:** 10.3762/bjnano.16.37

**Published:** 2025-03-31

**Authors:** Joy Mukherjee, Safiul Alam Mollick, Tanmoy Basu, Tapobrata Som

**Affiliations:** 1 SUNAG Laboratory, Institute of Physics, HBNI, Sachivalaya Marg, Bhubaneswar - 751 005, India; 2 Rabindra Mahavidyalaya, University of Burdwan, Hooghly, West Bengal, 712 401, India; 3 Centre for Quantum Engineering, Research and Education, TCG Centers for Research and Education in Science and Technology, Kolkata, West Bengal, 700 091, India; 4 Homi Bhabha National Institute, Training School Complex, Anushaktinagar, Mumbai - 400 085, India

**Keywords:** optimization of ion current, surface topography, TEM, ultralow-energy ECR-based ion source, UV–vis spectroscopy

## Abstract

This paper presents a comprehensive optimization of key parameters for generating ion beams in a microwave-coupled plasma-based ultralow-energy electron cyclotron resonance ion source, generally used for nanostructuring solid surfaces. The investigation focuses on developing, accelerating, and extracting Ar ions from a magnetron-coupled plasma cup utilizing a three-grid ion extraction composed of molybdenum. The study systematically examines the dependence of ion beam current on critical parameters, such as gas pressure, magnetron power, extraction voltage, and ion energies. The Gaussian nature of the beam profile is scrutinized and elucidated within the context of grid extraction-based ion sources. Plasma physics principles are employed to interpret the observed variations in the beam current with various parameters. The optimized beam current is used to investigate the inert ion-induced nanopatterning of silicon surfaces, at various ion fluences and incidence angles. The pre- and post-bombardment changes in optical properties, resulting from nanopatterned surfaces, are investigated using UV–vis reflectivity measurements and correlated with the dimensions of the nanopatterns. This manuscript highlights the potential applications arising from these findings, emphasizing the transformative impact of nanopatterning through low-energy inert ions.

## Introduction

Ion sources serve as fundamental components in numerous scientific and industrial applications and play a crucial role in generating charged particles. Various systems harness energetic ions for diverse purposes, spanning material science, high-energy physics, medical applications, and agricultural science [1–5]. Presently, energetic ions find application in various surface treatments such as nanopatterning, sputter etching, and controlled defect formation [6–7]. Particularly, ultralow-energy ion beams are exceptionally valuable for the precise modification of 2D layers [8] and ion-induced nanopatterning of semiconductor surfaces [9]. Over the past few decades, ion-induced nanopatterning and nanoscale functionalization have garnered significant interest, owing to their broad applications in DNA origami [10], tuning of wettability [11] and electrical and magnetic anisotropy [12–13], isolated dot formation [1], nanoscale plasmonic arrays [14], and field emission [15]. Thus, ion sources generate enormous possibilities for material modifications both physically and chemically. Further, there are diverse ion production mechanisms. The fundamental process of producing ions is the collision of atoms with ions or electrons, which may be either elastic or inelastic. In elastic collisions, the internal energy of the colliding particles does not change. Ionization, stripping, electron capture, and excitation of atoms due to collisions are examples of inelastic collisions. Free electrons colliding with atoms also produce ions. Electrons in the gas are heated by the inductively coupled method and then acquire enough energy to generate a plasma. Because of several drawbacks, such as Townsend discharge [16], these sources are not used nowadays. Compact broad-beam ion sources are widely used in scientific laboratories to generate ions. Depending upon the mechanism of production of various ions using gaseous plasma, the ion sources can be classified in direct current (DC)-operated ion sources, radio frequency discharge ion sources, and microwave-based electron cyclotron resonance (ECR) ion sources, as well as electron bombardment, charge exchange, and laser-driven ion sources [17–18]. In the past few decades, DC ion sources were commonly used [19–21]. These DC ion sources consist of a hot cathode or filament, which is not appropriate in cases of reactive gas discharge; hence, their lifetime is limited [22–23]. Moreover, the beam current produced by those ion sources is not suitable for modern-day applications. In material science as well as surface science applications, the ion source should be mobile and adaptable to the vacuum system, having a longer lifetime. Further, the ion source should produce a relatively high beam current (i.e., capable of forming a high density of plasma) with lower maintenance. To address this challenge, ECR-based ion sources were developed [24–25]. ECR ion sources are one of the most preferred ion sources for the easy production of ions with different energies and charge states. Since the discharge is maintained in the quartz cup via a strong electric field generated in the cavity, the ECR-based ion sources equipped with microwave cavities neither contain any filament nor any type of electrode [26]. The high plasma density within a quartz cup is confined by solenoid magnets surrounding it, creating a multi-cusp magnetic field. However, careful attention is required for the microwave coupling to the plasma cup to minimize the reflections of microwave power. Mechanical adjustments to the resonator length and waveguide are made to ensure minimal reflection. Additionally, maintaining the necessary magnetic field strength is crucial for sustaining the plasma. The ion source’s compact design is user-friendly and capable of producing a high beam current density using single- or multigrid extraction systems [27–28]. The extracted beam current is influenced by magnetron power, gas pressure, and extraction voltage. Furthermore, the beam current varies with different ion energies [29–30].

This article focuses on optimizing the beam current generated by a cost-effective microwave-based ECR ion source and the subsequent development of nanoscale patterns on the surface of silicon. The relationship between the beam current and various parameters is extensively examined and elucidated. Experimental parameters, spanning from plasma generation to ion beam extraction, are systematically optimized for the study of low-energy Ar-ion-induced nanostructures on silicon. The dependence of the extracted ion beam on gas pressure, magnetron power, and extraction grid voltage is documented for different ion energies. Additionally, the manuscript establishes the relationship between ion beam current and ion energy. Irradiation of p-type single crystal Si(100) surfaces at off-normal angles (60° and 72.5°) with 450 eV Ar ions results in the formation of well-defined nanoscale ripple patterns. The prominence of ripple structures increases with prolonged irradiation time, while bombardment at 72.5° with the same ion beam parameters leads to the coarsening of nanostructures. Cross-sectional transmission electron microscopy (TEM) measurements confirm the formation of nanostructures as observed from atomic force microscopy (AFM) images. The thickness of the amorphous thin layer is in good agreement with Monte Carlo simulations (SRIM) [31]. The article further investigates and explains the optical response (by UV–vis spectrometry) of the nanopatterned surfaces depending on the dimensions of the nanopatterns (i.e., wavelength and rms roughness). The potential applications of such nanopatterned silicon surfaces are highlighted. This article underscores the versatility of an optimized broad-beam ultralow-energy ion source, specifically in the context of optimization of inert Ar-ion beam and subsequent ion-induced silicon nanopatterning.

The TEM used for this work is a FEI Tecnai G2 12 Twin model, which operates at a voltage range of 20–120 kV. It employs a LaB_6_ emitter as the electron source and offers a line resolution of 0.2 nm with a maximum eccentric tilt angle of ±70°. Sample preparation for cross sectional TEM measurement involved mechanically grinding the substrate into a circular disk with a diameter of 3 mm and a thickness of approximately 100 μm. The disk was then subjected to dimpling to achieve uniform thinning. To further reduce the thickness to less than 40 μm at the center, ion milling was performed. This ultrathin central region was used for detailed TEM analysis.

## Description of the Ion Source

**Figure 1:**
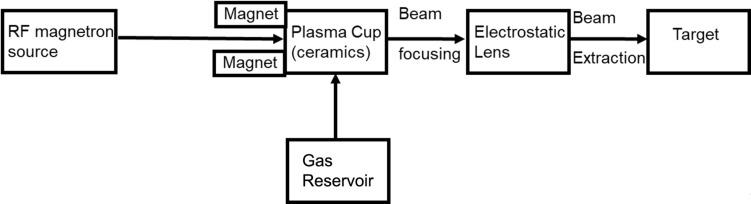
Block diagram of the components of the high-vacuum plasma ion source.

Figure 1 illustrates the block diagram of the magnetron-coupled ultralow-energy ECR ion source (Plasma Gen-II, Tectra GmbH, Germany). The schematic representation in Figure 1 elucidates the process of extracting an ultralow-energy ion beam. A magnetron is connected to the ceramic Al_2_O_3_ plasma cup via a waveguide. The gas inlet system facilitates the filling of the plasma cup with gas through a capillary tube. The intense electric field generated by the magnetron induces gas breakdown (discharge), leading to the formation of a highly intense plasma. The produced plasma is confined and sustained by a permanent magnet positioned near the plasma cup. For the extraction and focusing of the beam, a gridded electrostatic einzel lens is employed. The shape and size of the beam are contingent on the extraction voltage applied at the grid and the corresponding ion energy. The directed beam impacts the silicon target kept in ultrahigh vacuum (UHV) within the target chamber. A Faraday cup, connected to a multimeter, measures the beam current, and the corresponding ion fluence is expressed in terms of irradiation time. The sample holder located in a UHV chamber is connected to a five-axes (*x*, *y*, *z*, θ, φ) manipulator (PREVAC Technologies) system, offering movement and rotation in all possible directions. The sample is transferred to the ion source using a load-lock system.

**Figure 2:**
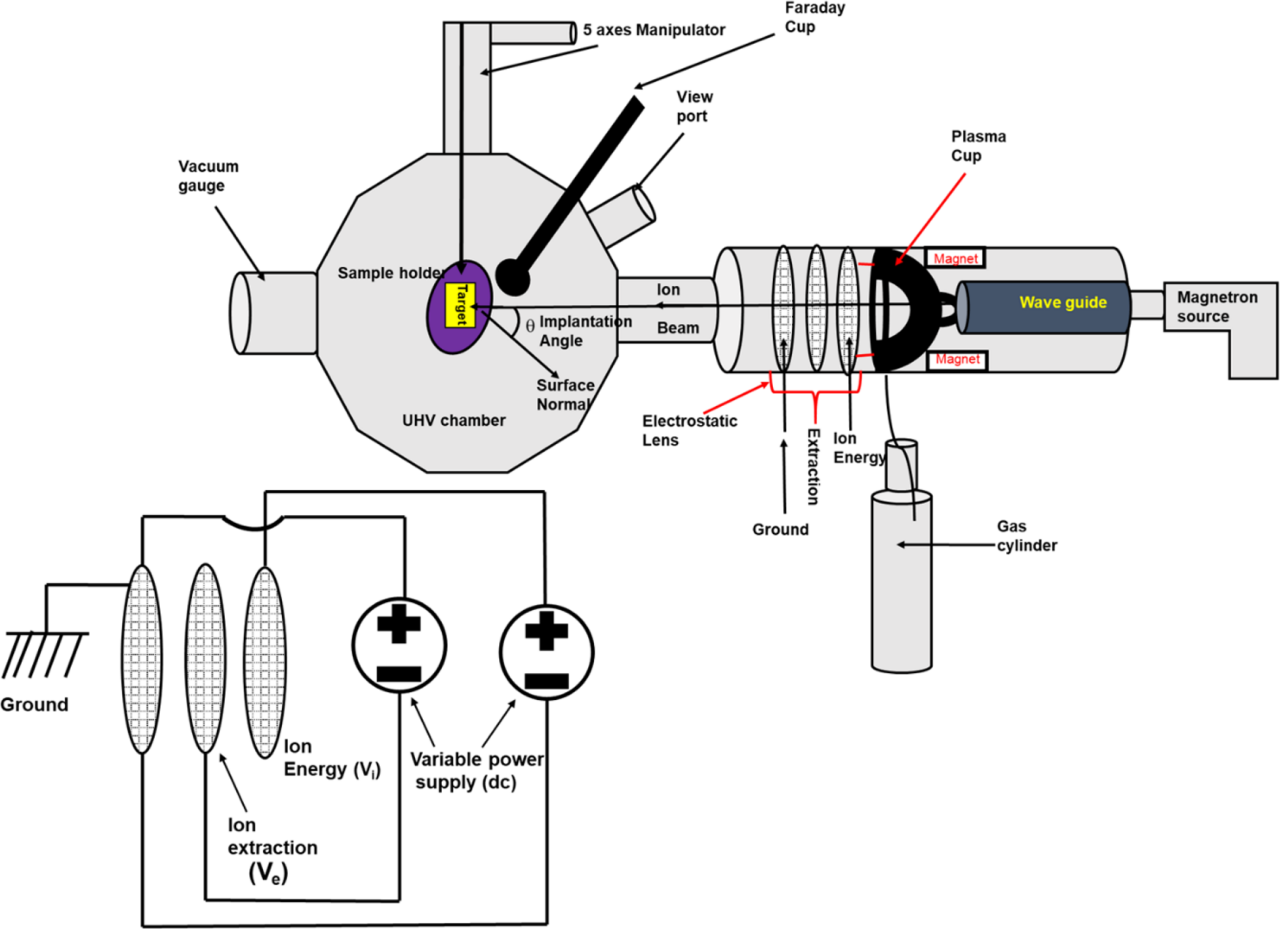
Cross-sectional schematic view of the microwave-coupled ultralow-energy ion beam system.

The cross-sectional view of the setup is shown in Figure 2. The type of magnetron-coupled ion source used here was first developed by Anton and coworkers [25]. The ion source is fitted in the cylindrical cavity of the UHV target chamber. The inner diameter of the plasma cup is around 52 mm. The cup is surrounded by water-cooled NdFeB magnets, which produce a multi-cusp field to confine the plasma. The 2.45 GHz magnetron microwave source is attached to the back side of the ion source, as shown in Figure 2. The dimension of the cylindrical resonator (waveguide) is chosen in such a way that it can produce maximum beam current. To generate plasma in the plasma cup, a gas is inserted into it through a capillary tube attached to the gas cylinder (reservoir). A pressure of 10^−4^ mbar is maintained for sustaining plasma by adjusting a needle valve attached to the gas reservoir. The entire length of the ion source is around 130 cm. The extraction of ion beams is accomplished by a three-grid ion optics system, as seen in Figure 2. The extraction voltage is applied to the grid to enable the extraction of an intense beam with different diameters. The circular perfection of the beam shape is evident from observations on the front plate attached to the UHV chamber. In this configuration, the beam current, specific to a given ion energy, can be finely adjusted based on magnetron power, working pressure, and extraction voltage. Additionally, the beam current is influenced by the extracted ion energy and the position of the target. Hence, a comprehensive investigation into the intricate relationship between ion current and the mentioned parameters emerges as a compelling topic in the current scientific context.

## Beam Extraction Grid

### Grid 1 (anode)

The anode, in contact with the plasma within the isolated plasma cup, serves to shift the plasma potential. By applying a voltage (e.g., +500 V) to the anode, the plasma potential is elevated to +500 V (plus the intrinsic plasma potential). When the sample is maintained at ground potential, the positive ions within the plasma are accelerated towards the sample with an energy approximately equal to the applied voltage.

### Grid 2 (extractor)

This grid is employed to control the divergence of the ion beam. A negative voltage is applied to the extractor grid, creating an electric field that influences ion trajectories. Additionally, a small negative field can extend from the extractor grid into the positively charged plasma region. This increases the ion extraction efficiency by enhancing the electric field gradient and enlarging the effective extraction volume.

### Grid 3 (grounded grid)

The third grid is maintained at ground potential and is particularly effective for operations involving very-low-energy ions. This grid enables ions to decelerate and traverse a field-free region near the source, ensuring minimal perturbations to the ion beam and facilitating precise ion transport.

## Results and Discussion

### Optimization of ion current through various plasma parameters

**Figure 3:**
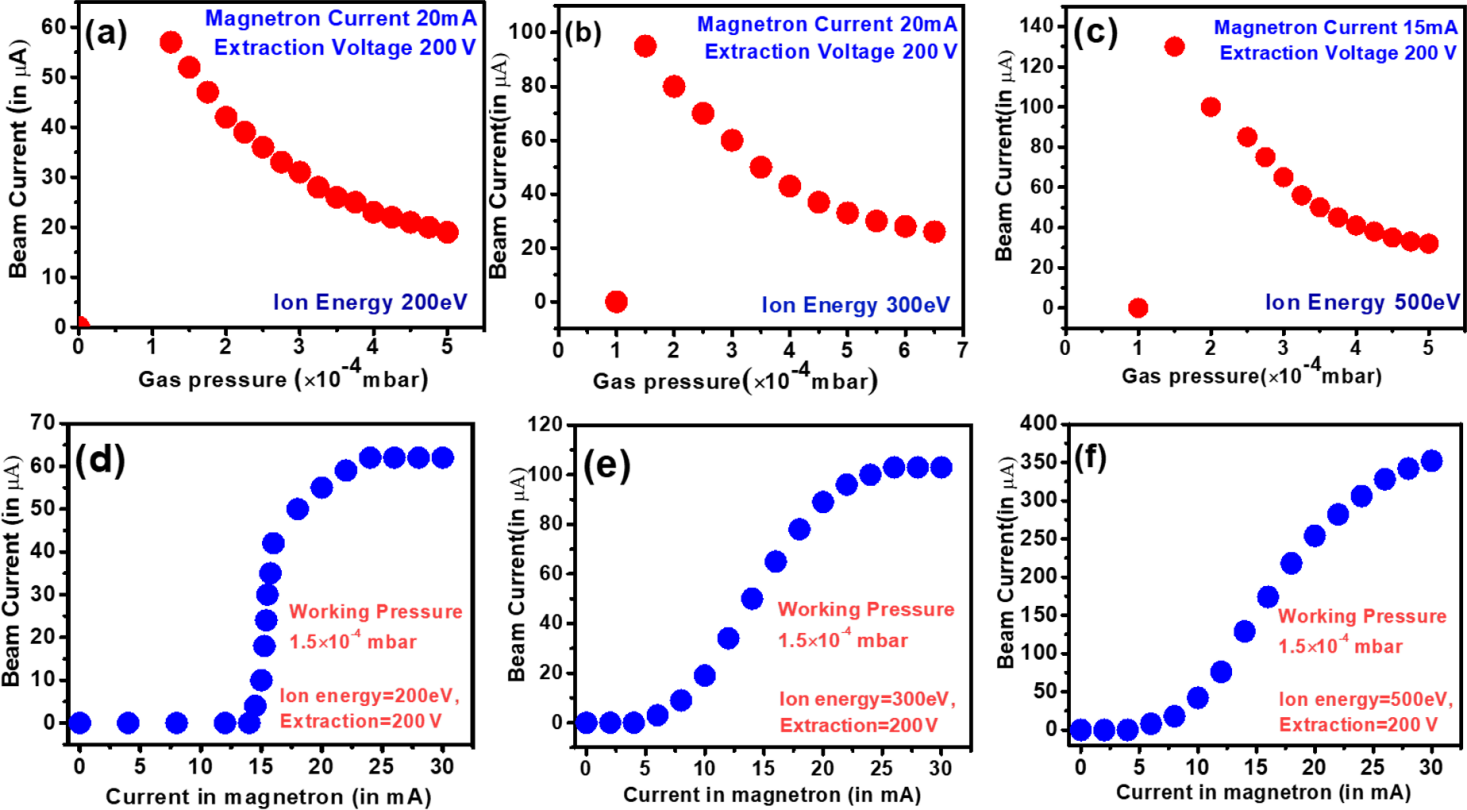
Variation of beam current with (a–c) gas pressure and (d–f) magnetron power for different ion energies.

The variation of beam current with gas pressure and magnetron power for different ion energies are investigated and presented in Figure 3. Figure 3a–c demonstrates that the beam current decays almost exponentially with the increase in gas pressure. The ion current is maximum at a gas pressure of 1.5 × 10^−4^ mbar, regardless of the ion energy. It is also evident that for the same gas pressure, the beam current is maximum for the highest ion energy. At low gas pressure, the mean free path of gas molecules is larger because of the lower density of gas molecules, which allows the produced ions to traverse a longer distance without collision. This increases the ionization efficiency, and hence, with fewer collisions, the probability for recombination of the ions is very low. Consequently, a large number of ions are extracted, intensifying the beam current. The entire phenomenon can be summarized through the equation λ = (σ·*n*)^−1^, where λ is the mean free path of the ions, σ is the recombination cross section, and *n* is the density of the ions inside the plasma [32–34]. The mean free path of the ions, determined by the recombination cross section and density of plasma, plays a key role in quantifying the ion current.

Further, the conversion of the gas to plasma is governed by a magnetron source; therefore, the ion current or plasma density depends on magnetron power. To understand that, the variation of ion current with microwave power is recorded at different gas pressure and ion energies, as presented in Figure 3d–f. In general, the plasma density (*n*) depends on the microwave frequency (ω) as *n* = *E*_RF_ω^2^/ε, where *E*_RF_ is the microwave power and ε is the minimum energy required for ion–electron pair generation [32]. The magnitude of ε is different for different gases. It is evident from Figure 3d,e that up to a critical microwave power, no plasma is formed, resulting in a zero beam current. With the increase of magnetron power beyond ε, the beam current increases almost linearly with the input magnetron power since *n* is directly proportional to *E*_RF_. The beam current reaches saturation at a specific microwave power level, which varies based on the ion energy. (Figure 3d–f). Further, the cutoff power also depends on the ion energy. At low ion energies, the microwave power required for generating a plasma is high. With higher ion energies, the microwave power required for ion–electron pair generation decreases.

**Figure 4:**
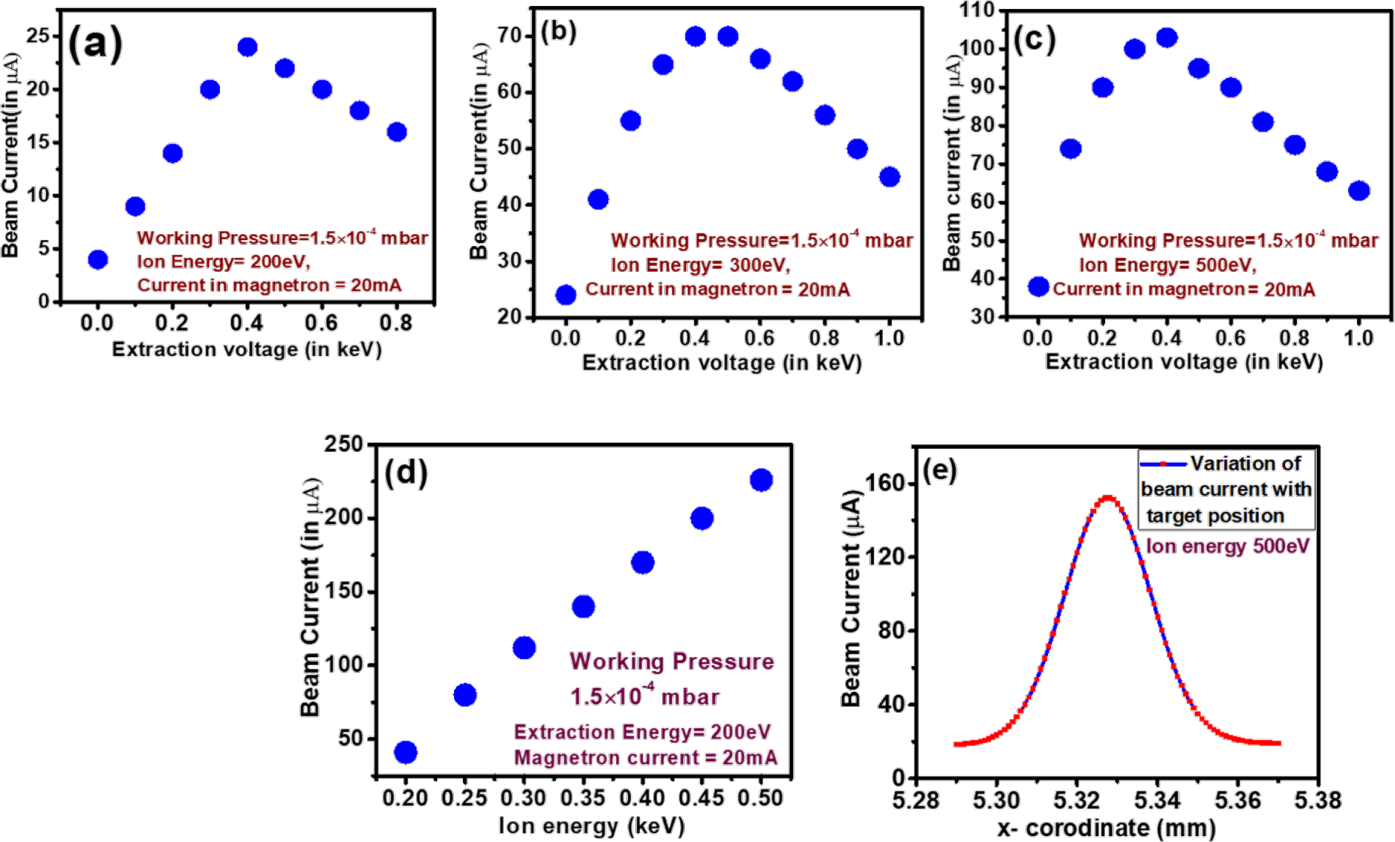
Variation of (a–c) beam current with ion extraction voltage at different ion energy; (d) beam current as function of the ion energy; (e) beam current as function of the target position.

The Ar-ion beam is extracted via a three-grid ion optics system [35–37]. The beam current and the beam profile depend on the potential applied at the grid and the target. The change in beam current with the ion extraction voltage recorded for different ion energies is presented in Figure 4a–c. Initially, the beam current increases linearly with the applied extraction voltage since more ions are extracted at higher extraction voltages. Regardless of the ion energy, the beam current is maximum at an extraction voltage of 400 V. With further increases in extraction voltage, the beam current decreases rapidly. Beyond this threshold of 400 V, the increased extraction voltage induces significant defocusing of the ion beam. This highlights the critical role of the extraction voltage in maintaining beam coherence. Generally, the extraction voltage is kept fixed to maintain the shape of the beam, essential for uniform irradiation of samples.

The dependence of beam current on the ion energy is given in Figure 4d. The beam current increases almost linearly with increasing ion energy at a fixed extraction voltage and microwave power. For a particular ion energy, lowering the extraction voltage also results in a lowering of beam current as observed from the above Figure 4a–c. Therefore, to maintain a proper beam shape and adequate beam current, the extraction voltage and ion energy are to be precisely optimized. Further, the variation of beam current with the target position, known as the beam profile, is also presented in Figure 4e. The beam profile is Gaussian for concave grid beam extraction optics.

### Nanostructuring on Si surface by 450 eV Ar-ion bombardment

**Figure 5:**
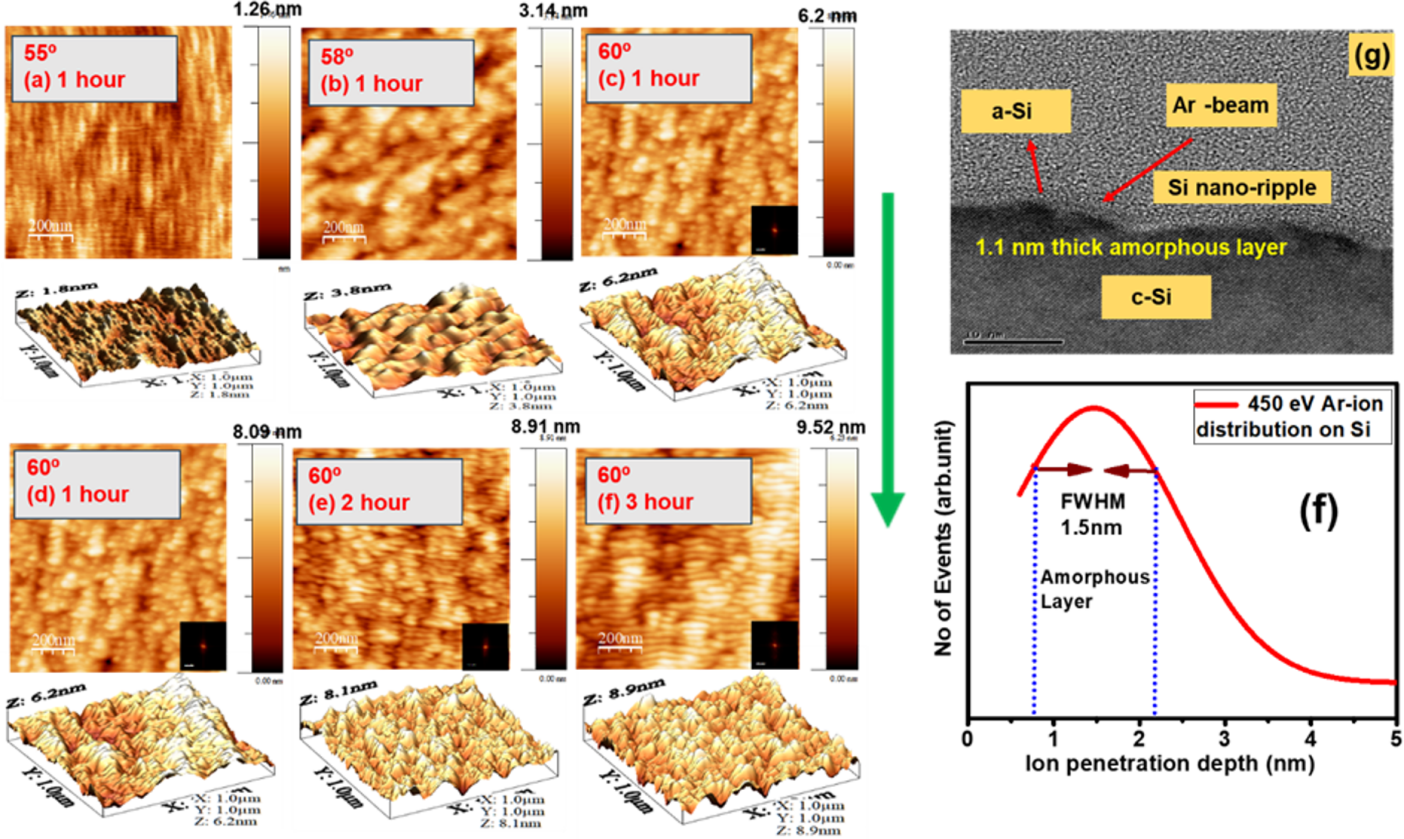
AFM image (2D and 3D) of the evolution of surface morphology after 450 eV Ar-ion bombardment at different incidence angles and irradiation times. The arrow indicates the ion beam direction.

The morphological evolution of Si after the off-normal bombardment with 450 eV Ar ions at different incidence angles and for various irradiation times is investigated using AFM in tapping mode. Si cantilevers with tip radii of 10 nm were employed, with scan rate of 1 µm/s and a fixed scan size of 5 µm × 5 µm. Quantitative analysis of the surface topography was conducted using WSxM software. Figure 5 presents the surface morphology of the Si surface after Ar-ion bombardment at different incidence angles. The arrow on the right-hand side indicates the direction of the ion beam concerning the surface normal. The irradiation of the silicon surface at an angle of 55° leads to no changes in surface morphology (Figure 5a). However, at an ion incidence angle of 58°, changes in surface morphology begin to appear, although no prominent ripple structure is observed (Figure 5b). In contrast, the bombardment of the Si surface for 1 h at an angle of 60° leads to the formation of a well-defined nanoscale ripple pattern as observed in Figure 5c. The growth of the ripple becomes more prominent with the increase in bombardment time, that is, the amplitude of the ripples grows.

**Figure 6:**
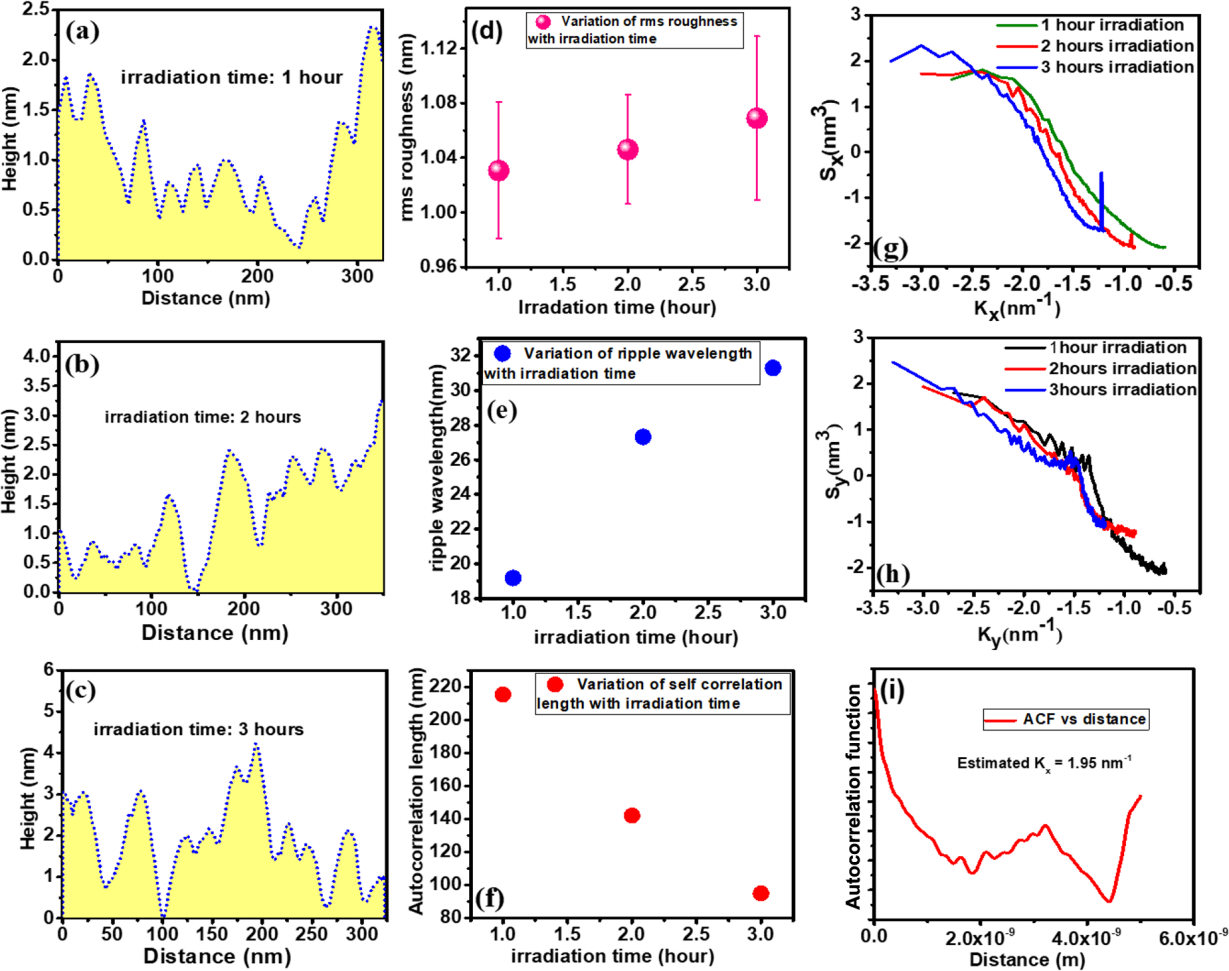
Variation of (a–c) surface height modulation of the nanopatterned silicon surface; (d) ripple wavelength and (e) rms roughness as function of the irradiation time. Variation of power spectral density of the nanopatterned silicon surface in (g) parallel and (h) perpendicular direction. (f) Variation of autocorrelation length with irradiation time. (i) Variation of the autocorrelation function with distance.

To visualize the growth of the ripples, 3D AFM images are presented along with 2D images. The ripple height increases with bombardment time. Fast Fourier transform (FFT) images of the nanopatterned surface are inset in the lower right corner of each image. In the present case, the fluence is represented by irradiation time. The quality and the growth of the nanostructures are quantitatively discussed in Figure 6, where variations of ripple wavelength, rms roughness, and power spectral density are discussed.

Figure 5g shows the cross-sectional TEM image after 450 eV Ar-ion bombardment of the Si surface at an angle of 60° for a time of 3 h. The presence of Ar-ion-induced surface corrugation in terms of ripple-like nanostructures is evidenced in Figure 5g. Although the amplitude of the ripples is not large, the observed ripple wavelength of around 31 nm from the TEM image is consistent with that of AFM data (Figure 6e). However, in addition to the ripple-like nanostructures, an ultrathin amorphous layer is formed because of the Ar-ion bombardment. The thickness of the amorphous layer is around 1.5 nm, which is consistent with the penetration depth of the Ar ions (1.2 nm) estimated by Monte Carlo simulations (Figure 5f) [31]. Therefore, the topographical image is consistent with the cross-sectional image, indicating a clear signature of ripple-like nanostructure formation.

Figure 6a–c shows the variation of the surface profile of the AFM images shown in Figure 5. The height profile is direct evidence of the variation of ripple amplitude with irradiation time. The increase in ripple height with irradiation time is shown in Figure 6a–c. Further, the fluctuation in ripple height or amplitude, generally termed as rms roughness, is also investigated in Figure 6d. The rms roughness increases linearly with the irradiation time (i.e., fluence). Further, the ordering of the nanostructure with bombardment time, examined in terms of ripple wavelength, is presented in Figure 6e. The ripple wavelength increases as the bombardment time increases from 1 to 2 h. With a further increment in irradiation time, the change of ripple wavelength is negligible, that is, a saturation of ripple wavelength is observed. The degree of similarity between two spatial morphologies is generally quantified by the autocorrelation length, as presented in Figure 6f. The autocorrelation length decreases with bombardment time. This indicates that less ordered ripple structures develop with higher irradiation times. To understand the growth of the ripple structure, the power spectral density factor along the parallel and perpendicular direction of the developed ripples is presented in Figure 6g,h. The prominent peak present in Figure 6g indicates the development of the ripple structure along the *x* direction (parallel) with a particular wavevector (*k_x_*). The absence of a ripple wavevector in the perpendicular direction is evidenced in Figure 6h. The estimated *k_x_* from the autocorrelation function (Figure 6i) is almost consistent with the one obtained from the power spectral density (Figure 6g). Thus, 450 eV Ar-ion bombardment on Si leads to the formation of well-defined parallel ripples at off-normal incidence.

**Figure 7:**
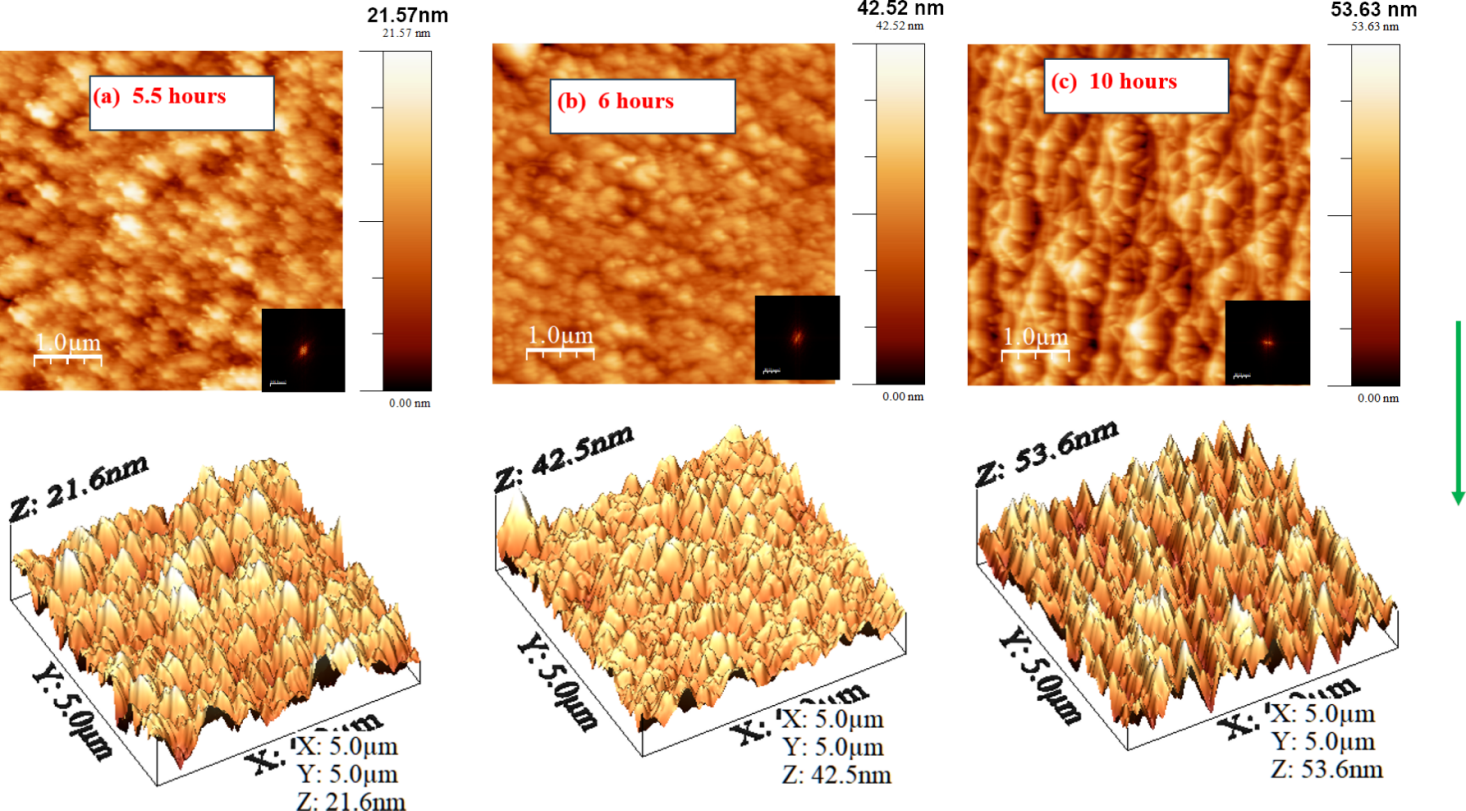
AFM images (2D and 3D) of the evolution of nanostructures on the Si surface with irradiation time at an angle of 72.5°.

Figure 7 illustrates the surface topography after 450 eV Ar-ion bombardment of the silicon surface at an angle of 72.5° as function of the bombardment time. 3D AFM images are presented along with 2D surface topography images. Generally, the transformation of well-defined nanoripples into nanofacets is expected at such near-grazing-incidence irradiation [38]. In the present case, although no prominent nanofacet formation is observed, a clear signature of the transformation of ripple structures into nanofacets is seen. After a sufficiently large bombardment time (10 h), nanofacet-like structures with larger dimensions develop, although the facets are not well organized. It is also evident that the rms roughness increases with bombardment time.

Surface nanostructuring by energetic ion bombardment is a consequence of ion-beam-induced off-normal (60° and 72.5°) sputtering of surface atoms and their consecutive redistribution [9,39–40]. During ion bombardment, the unequal radius of curvature of the surface leads to unequal deposition of energy at different points on the surface, which results in unequal sputtering at those points. This generates surface instabilities, and consequently, the surface atoms are redistributed to stabilize the surface. These two effects jointly trigger nanopattern formation on the surface. A first theoretical model was proposed by Bradley and Harper [41], based on curvature-dependent sputtering of surface and near-surface atoms. Later, Carter and Vishnyakov introduced the concept of redistribution of surface atoms [42]. Several experiments have been carried out to understand other factors that contribute to nanopattern formation, such as preferential and differential sputtering [6,43], the role of surface and beam impurities, and the effect of chemical compound formation and compound ion irradiation [44–45]. In cases of ultralow-energy ion bombardment, the rate of sputtering is lower compared to medium-energy ion bombardment; therefore, in this case, mass redistribution of the surface atoms plays a key role. There is no reaction between the inert Ar ions and the Si atoms, ensuring the absence of a chemical aspect of pattern formation. However, the native silicon oxide layer is partially sputtered. This is also a key factor in generating surface instabilities. The surface morphology largely varies due to different extents of near-surface mass transport by the surface-confined ion-enhanced viscous flow [46]. Here, up to an ion incidence angle of 58°, the surface becomes unstable under 450 eV Ar-ion bombardment. Due to sputtering, a well-defined ripple formation is found after 1 h of 450 eV Ar-ion bombardment. With the increase in bombardment time, more silicon and oxygen atoms are sputtered. Due to the presence of the ripples, the surface becomes anisotropic. The consequence of such an anisotropic nature of the surface is investigated and discussed in the upcoming section.

### Application of nanopatterned Si surface

**Figure 8:**
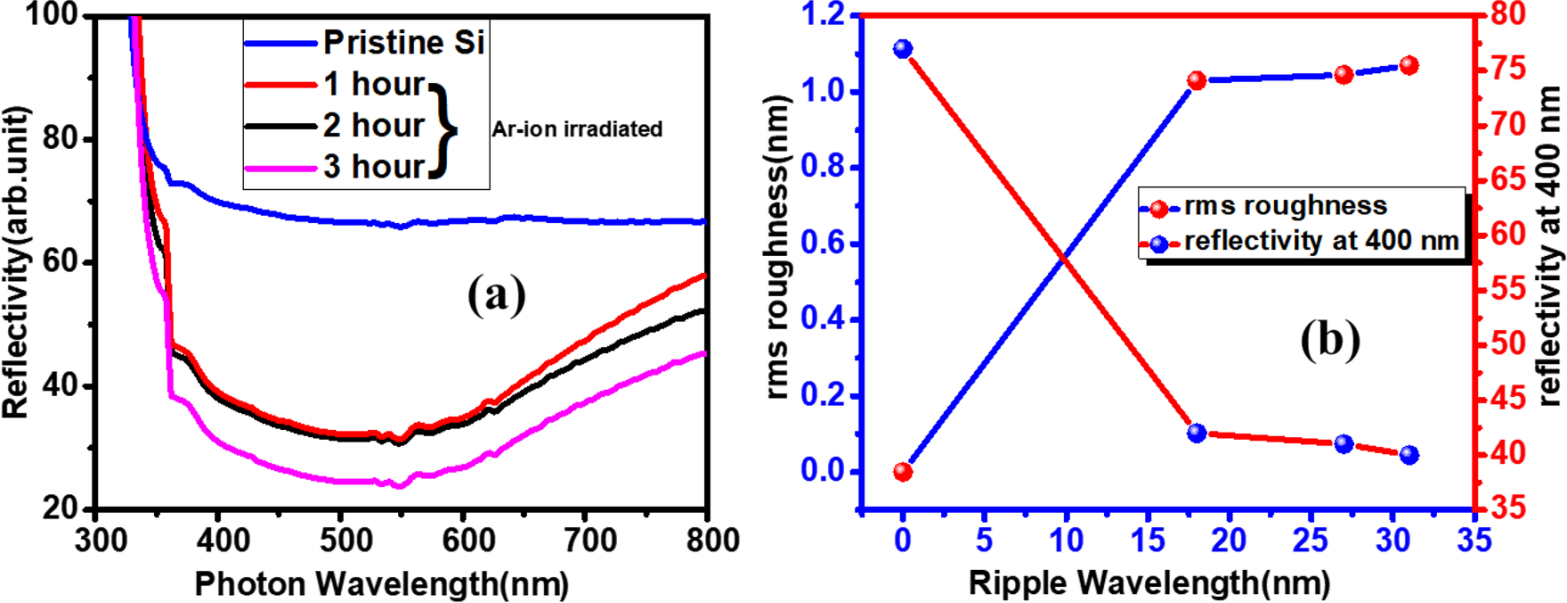
(a) UV–vis spectra of pristine and nanopatterned surfaces, (b) variation of rms roughness and reflectivity with ripple wavelength.

The optical response of pristine and Ar-ion-induced nanopatterned silicon surfaces are investigated through UV–vis reflectivity measurements and presented in Figure 8. Figure 8a depicts the change in reflectivity of the silicon surface due to the presence of nanopatterns. With the increase of bombardment time, reflectivity decreases drastically. The change in reflectivity with respect to rms roughness and ripple wavelength is shown in Figure 8b. It is clear from Figure 8b, that the reflectivity decreases with the increase in ripple wavelength. In general, the presence of nanopatterns on the surface reduces the reflection of UV–visible light because of light trapping by multiple reflections [47–49]. Ar-ion bombardment for 3 h leads to the development of a well-defined nanopattern on the silicon surface; hence, the reflectivity becomes minimal compared to the other two surfaces that were irradiated for shorter periods of time. The change in reflectivity depends on the change in the electronic structure as well as surface topography of the material. A change in electronic structure can be related to changes in chemical nature, impurity incorporation on the surface, and amorphization of the surface. Inert Ar causes no chemical modifications of the silicon surface. Also there are no implanted Ar ions on the silicon surface (Figure 5g), particularly in this lower-energy regime. Therefore, in the present case, the amorphization due to ion beam sputtering and the nanostructure formation change the electronic density of the material, causing a lowering in reflectivity. The tailoring of the reflectivity by developing nanostructures is widely applicable for anti-reflective coatings and photovoltaic device applications [50–51].

The formation of nanostructures on the silicon surface by inert-ion bombardment is a consequence of ion-induced instabilities on the surface by the interplay between sputtering and mass redistribution of surface atoms [52–53]. During ion bombardment, the sputtering of the native silicon oxide layer along with that of bulk silicon takes place. The rate of sputtering of silicon oxide and the elemental silicon is different, which leads to instabilities during bombardment and the development of nanopatterns on the surface. Further, the exposure of the nanopatterned silicon surface to air during optical measurement ensures the formation of non-uniform silicon oxide on the nanopatterned silicon surface. Additionally, the post-bombardment growth of silicon oxide on nanopatterned silicon leads to site-dependent growth of native oxide, which is useful for producing hysteresis in surface current–voltage characteristic measurements [50–51]. The preferential spatial formation of silicon oxide changes the reflectivity. Also, nanopatterned silicon surfaces can be an alternative for memory devices.

## Conclusion

In this manuscript, the intricacies of an ultralow-energy magnetron-based electron cyclotron resonance (ECR) ion source are studied systematically by exploring optimal parameters to achieve stable and intense beam currents. The cost-effectiveness and versatility of this ion source make it particularly noteworthy, offering a practical solution for generating reasonable beam currents. The ion source operates within an ultrahigh-vacuum environment, rendering it valuable for both implantation and deposition processes. Our meticulous investigation of the ECR ion source lays the groundwork for ion beam-induced nanostructuring and layer-wise material modification, affording precise control over ion penetration depth and fluence. The manuscript emphasizes an intriguing alternative perspective by highlighting the in-depth optimization of the ion source and inert ion-induced nanopatterning as a viable approach for anti-reflective coatings. This study not only advances our understanding of ECR-based ion sources but also opens avenues for innovative applications in nanotechnology and materials science.

## Data Availability

Data generated and analyzed during this study is available from the corresponding author upon reasonable request.
